# Neonatal Morbidities and Hospitalization in the First 2 Years of Life Among Infants Born Very Preterm

**DOI:** 10.1001/jamanetworkopen.2025.30123

**Published:** 2025-09-03

**Authors:** Tim J. van Hasselt, Rebecca A. Dorner, Anup Katheria, Cheryl Battersby, Chris Gale, David K. H. Lo, Sarah E. Seaton

**Affiliations:** 1 Department of Population Health Sciences, University of Leicester, Leicester, United Kingdom; 2 Neonatal Research Institute, Sharp Mary Birch Hospital for Women & Newborns, San Diego, California; 3 School of Public Health, Faculty of Medicine, Imperial College London, London, United Kingdom; 4 Department of Respiratory Sciences, University of Leicester, Leicester, United Kingdom

## Abstract

**Importance:**

Children born very preterm have increased health care use. However, there is a lack of research using contemporary national data quantifying hospitalizations after neonatal discharge to inform counseling of families and health care provision.

**Objective:**

To examine hospital admissions after neonatal discharge and before 2 years of age among children born at less than 32 weeks’ gestation and assess associations between hospitalization and neonatal morbidities.

**Design, Setting, and Participants:**

This retrospective cohort study used data from all neonatal units and admitting hospitals in England and Wales supplied from the National Neonatal Research Database, linked with the Hospital Episode Statistics Admitted Patient Care database in England and the Patient Episode Database for Wales. Participants were children born at 22 through 31 weeks’ gestation from January 1, 2013, to December 31, 2018, who were admitted to neonatal units and discharged home. Analysis was performed from June 26, 2024, to June 3, 2025.

**Exposures:**

Gestational age; sex; small-for-gestational-age status; season at time of neonatal discharge; neonatal morbidities, including bronchopulmonary dysplasia (BPD), severe necrotizing enterocolitis (NEC), and neonatal brain injury; and number of morbidities.

**Main Outcomes and Measures:**

Prevalence of hospital admission and total calendar days hospitalized across all admissions. Negative binomial regression was used to calculate the adjusted incidence rate ratio (AIRR) for total calendar days hospitalized, adjusting for gestational age, sex, small for gestational age, and season of neonatal discharge.

**Results:**

Among the 39 413 children included (21 360 [54.2%] male; median gestational age at birth, 29 weeks [IQR, 27-31 weeks]), 26 276 (66.7%) did not have major neonatal morbidities. A total of 26 498 children (67.2%) had at least 1 episode of hospitalization between neonatal discharge and the age of 2 years. This ranged from 6138 of 10 444 children born at 31 weeks’ gestation (58.8%) to 450 of 517 born at less than 24 weeks (87.0%). The median number of total calendar days hospitalized across admissions increased from 1 day (IQR, 0-5 days) for children born at 31 weeks’ gestation to 8 days (IQR, 3-21 days) for those born at less than 24 weeks. Neonatal morbidities were associated with increased total days hospitalized; the AIRR for total hospitalization days for children with vs without BPD was 1.80 (95% CI, 1.72-1.88), for those with vs without severe NEC was 1.88 (95% CI, 1.65-2.15), and for those with vs without neonatal brain injury was 1.46 (95% CI, 1.36-1.57). Combinations of morbidities were associated with total expected days hospitalized: the model estimated that a child born at less than 24 weeks’ gestation with 3 morbidities would have 40.6 days (95% CI, 34.8-44.3 days) of hospitalization before age 2 years.

**Conclusions and Relevance:**

In this cohort study of children born very preterm discharged from neonatal care in England and Wales, most children experienced hospitalization before their second birthday, and hospitalizations were associated with lower gestational age and neonatal morbidities. These findings can aid counseling and suggest that further research should investigate interventions to prevent hospitalization in this population.

## Key Points

**Question:**

What is the proportion of children born very preterm in England and Wales who are hospitalized between neonatal discharge home and the age of 2 years?

**Findings:**

In this cohort study of 39 413 children born at less than 32 weeks’ gestation and discharged home from neonatal care, 67% had at least 1 episode of hospitalization before the age of 2 years, mostly for respiratory illness.

**Meaning:**

Most children born very preterm experienced hospital admissions after initial discharge home; these data will inform family counseling and prevention efforts.

## Teaser

This cohort study examines the prevalence of hospital admissions before age 2 years among children born at less than 32 weeks’ gestation in England and Wales and associations between hospitalization and neonatal morbidities.

## Introduction

There is increasing survival following preterm birth, particularly at lower gestations.^1,2^ However, incidence of complications and later-life sequelae appears to be increasing in recent cohorts.^3,4,5^

Children born very preterm (<32 weeks’ gestation) also have greater health care use, including hospitalization, than term-born peers (≥37 weeks’ gestation).^6,7,8^ Hospitalization of preterm-born children after neonatal discharge has profound impacts on the whole family, both psychologically and economically.^9^ Considering days at home (rather than days attending health care settings) has been raised as an important outcome for families.^10^

Hospital admissions may relate to complications of prematurity, such as bronchopulmonary dysplasia (BPD) with subsequent respiratory infection^11^ or intraventricular hemorrhage (IVH) and subsequent ventriculoperitoneal shunting.^12^ Neonatal morbidities after preterm birth, such as BPD or brain injury (including severe IVH and sequelae), are associated with the adverse outcomes of mortality and neurodevelopmental impairment.^13^ Furthermore, the total number of severe neonatal morbidities can be used to estimate adverse outcomes^13^ and could serve as an important outcome in trials, using ordinal methods,^14^ as it is available at the point of neonatal discharge.

To our knowledge, no studies have quantified hospitalization after neonatal discharge of children born very preterm at a national level using contemporary data and explored its association with the number of neonatal morbidities. We aimed to use a novel linkage between routinely collected neonatal data and hospital admission data to assess the association between significant neonatal morbidities and subsequent total days hospitalized among children born very preterm. This could provide data for counseling families and may provide evidence to support the use of neonatal morbidities as a trial outcome.

## Methods

This cohort study used the National Neonatal Research Database (NNRD), which supplied data on all births at less than 32 weeks’ completed gestation in England and Wales from January 1, 2013, to December 31, 2018. We used routinely recorded data, and no participants were actively recruited. The East of England committee and the Confidentiality Advisory Group provided research ethical approval to use personal identifiers for the purpose of linkage, with a waiver of informed consent because all data were deidentified. We reported this study following the Strengthening the Reporting of Observational Studies in Epidemiology (STROBE) guideline for cohort studies.

The NNRD captures all neonatal unit admissions with complete coverage in England and Wales from 2013 and includes demographic and clinical data, which undergo data verification and cleaning.^15^ These were linked to data on all hospital admissions in England and Wales before the chronological age of 2 years from Hospital Episode Statistics Admitted Patient Care (APC) in England and the Patient Episode Database for Wales (collectively referred to as *APC data* hereafter). APC datasets provide routinely collected data on all National Health Service (NHS) hospital admissions since 1989 (England) or 1991 (Wales), including admission diagnoses (*International Statistical Classification of Diseases and Related Health Problems, 10th Revision* [*ICD-10*]) and dates of admission and discharge.^16,17^

Child-level linkage was performed by the NHS Digital (now NHS England) Informatics Service and NHS Wales Informatics Service using personal identifiers (NHS numbers, available for >99% of children, and also, in England, date of birth, surname, and postcode) with subsequent deidentification, including removing name, date of birth, and exact date of neonatal discharge (given only as month and year after deidentification). Linkage to data from the Office for National Statistics provided information on all deaths registered before the age of 2 years, although to avoid patient identification, dates of deaths were not provided.

We included children who were born between 22 weeks 0 days and 31 weeks 6 days of completed gestation, were admitted for neonatal care after birth, and survived to discharge home from neonatal care (excluding children transitioned to another health care setting, such as a pediatric ward or pediatric intensive care unit). Because children most frequently become unwell within the first years of life due to respiratory and immune system immaturity,^18,19^ we identified hospital admissions occurring after neonatal discharge and before 2 years of age from APC data, as well as the total calendar days hospitalized across these admissions (to reflect the reduction in days at home), as our primary outcomes. Multiple episodes of care involving different clinical teams during hospitalization were merged into spells (duration from admission to discharge). We excluded admissions classified as birth admissions from the admission source and admission method variables. In addition, where admissions took place within 1 month of the final neonatal discharge, we only included admissions from the usual place of residence in order to avoid misclassifications of transfers between neonatal units.

### Statistical Analysis

We compiled summary statistics for the cohort, presenting frequencies and percentages for categorical variables, means and SDs for normally distributed variables, and medians and IQRs for nonnormally distributed variables. For multivariable analysis, we performed negative binomial regression analysis using total calendar days hospitalized. Based on previous work considering number of morbidities as an epidemiologic or trial outcome,^13,20,21^ we examined the following significant neonatal morbidities as binary variables: BPD, defined as requiring respiratory support or oxygen use at 36 weeks’ postmenstrual age; severe necrotizing enterocolitis (NEC), confirmed at surgery^22^; neonatal brain injury, defined as grade 3-4 intraventricular hemorrhage, cystic periventricular leukomalacia, hydrocephalus, or meningitis^23^; and severe retinopathy of prematurity (ROP), defined as grade 3 or above and/or requiring treatment. Where there were considerable and comparable increases in the adjusted incidence rate ratio (AIRR), we created a count variable for the neonatal morbidities.

Based on previous research,^8,24^ we also adjusted for completed weeks of gestation (as a categorical variable due to nonlinear associations); sex; small for gestational age (SGA) status; and season of neonatal discharge: spring (March to May), summer (June to August), fall (September to November), and winter (December to February). We coded birth weights greater than 3 SDs from the median for gestational age and unknown or unreported sex as missing values^25^ and excluded those children from multivariable analyses. SGA was defined as less than the 10th centile of birth weight for gestational age and sex using existing thresholds.^26,27^ All statistical analyses were performed using Stata, version 18.0 (StataCorp LLC). Analyses were performed from June 26, 2024, to June 3, 2025. Two-sided *P* < .05 was considered significant, and results of multivariable analyses are presented with 95% CIs.

## Results

There were 40 690 children born at 22 weeks 0 days to 31 weeks 6 days of gestation from 2013 to 2018 in England and Wales who were discharged home. We excluded 1277 children (3.1%) who had no linkage to any record of hospitalization, including their neonatal stay; the proportion of children with no linkage by gestational age ranged from 377 of 10 821 (3.5%) born at 31 weeks’ gestation to 31 of 1416 (2.2%) born at 24 weeks. Of the remaining 39 413 children included (median gestational age at birth, 29 weeks [IQR, 27-31 weeks]; 18 030 [45.7%] female, 21 360 [54.2%] male, and 23 [0.0%] with missing sex), 26 498 (67.2%) had at least 1 hospitalization after neonatal discharge (with a total of 92 494 hospitalization spells), and 12 915 (32.8%) did not. Among hospitalizations, we excluded 1 linked hospital admission that was a complete duplicate across all variables. Of the children with at least 1 hospitalization, there were 28 (0.1%) who had linkage to APC but incomplete data for length of hospital stay. Data for these children were included in the initial descriptive analysis using hospitalization as a binary outcome but were excluded from multivariable analyses.

**Figure 1.**
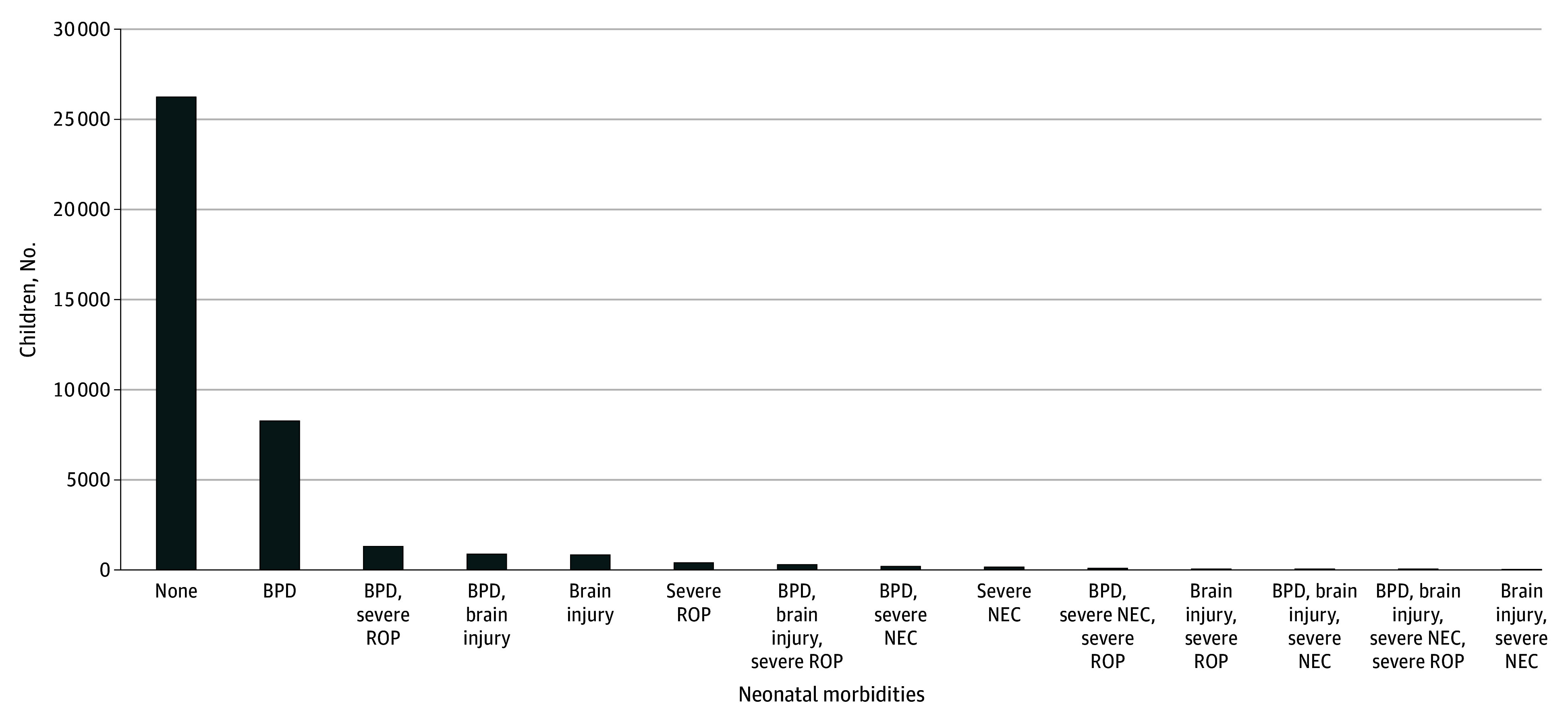
UpSet Plot of Morbidities of Bronchopulmonary Dysplasia (BPD), Neonatal Brain Injury, Severe Necrotizing Enterocolitis (NEC), and Severe Retinopathy of Prematurity (ROP) Among Individuals Discharged Home From Neonatal Care BPD was defined as requiring oxygen or respiratory support at 36 weeks’ postmenstrual age, severe NEC as NEC confirmed at surgery, and severe ROP as grade 3 or above and/or requiring treatment.

Most children in the overall cohort (26 276 [66.7%]) did not have BPD, severe NEC, severe ROP, or neonatal brain injury (Figure 1), and the next largest group had BPD alone (8306 [21.1%]), followed by BPD and severe ROP (1353 [3.4%]). There were few children with all 4 morbidities of BPD, severe NEC, severe ROP, and neonatal brain injury (55 [0.1%]).

Children with vs without hospitalization had a higher proportion of males (15 266 [57.6%] vs 6094 [47.2%]), a lower mean (SD) birth weight (1207 g [365.7 g] vs 1321 g [340.3 g]), and a higher proportion with neonatal morbidities (eg, 9098 [34.3%] vs 2231 [17.3%] had BPD) (Table 1). The proportion with congenital anomalies was greater among children hospitalized vs not hospitalized, although it was still low (600 [2.3%] vs 13 [1.0%]), and there were fewer children still with severe congenital anomalies requiring early surgical intervention: 146 children with hospitalization (0.6%) vs 35 (0.3%) without (using the definition by Helenius et al,^28^ including diagnoses such as duct-dependent congenital heart disease, congenital diaphragmatic hernia, and abdominal wall defects). Mortality between neonatal discharge and 2 years of age was low: 159 children with hospitalizations (0.6%) died, compared with 73 (0.6%) without hospitalizations (Table 1).

**Table 1.** Birth and Neonatal Characteristics of Children Born at Less Than 32 Weeks’ Gestation Between 2013 and 2018 in England and Wales With and Without Hospital Admission After Neonatal Discharge to Home

Characteristic	Children (N = 39 413)^a^	
	With hospital admission after neonatal discharge	Without hospital admission after neonatal discharge
Total children, No. (%)	26 498 (67.2)	12 915 (32.8)
Sex		
Female	11 212 (42.3)	6818 (52.8)
Male	15 266 (57.6)	6094 (47.2)
Missing data	20 (0.1)	3 (0.0)
Gestational age at birth, wk		
<24	450 (1.7)	67 (0.5)
24	1182 (4.5)	203 (1.6)
25	1594 (6.0)	385 (3.0)
26	2130 (8.0)	609 (4.7)
27	2702 (10.2)	924 (7.2)
28	3533 (13.3)	1417 (11.0)
29	3863 (14.6)	2045 (15.8)
30	4906 (18.5)	2959 (22.9)
31	6138 (23.2)	4306 (33.3)
Birth weight		
Mean (SD), g	1207 (365.7)	1321 (340.3)
Small for gestational age^b^	2374 (9.0)	807 (6.3)
Missing data	111 (0.4)	48 (0.4)
Multiple birth status		
Singleton	19 947 (75.3)	9165 (71.0)
Multiple	6551 (24.7)	3750 (29.0)
Antenatal steroids		
None	1233 (4.7)	700 (5.4)
Complete^c^	18 928 (71.4)	9016 (69.8)
Incomplete	4768 (18.0)	2405 (18.6)
Missing data	1569 (5.9)	794 (6.2)
Mode of delivery		
Vaginal	9308 (35.1)	4478 (34.7)
Instrumental vaginal	661 (2.5)	374 (2.9)
Cesarean	15 446 (58.3)	7520 (58.2)
Missing data	1083 (4.1)	543 (4.2)
Congenital anomalies		
Any present	600 (2.3)	133 (1.0)
Severe^d^	146 (0.6)	35 (0.3)
Season of neonatal discharge		
Spring (March-May)	6506 (24.6)	3292 (25.5)
Summer (June-August)	6766 (25.5)	3436 (26.6)
Fall (September-November)	6654 (25.1)	3026 (23.4)
Winter (December-February)	6572 (24.8)	3161 (24.5)
BPD^e^		
None	17 264 (65.2)	10 615 (82.5)
Present	9098 (34.3)	2231 (17.3)
Missing data	136 (0.5)	69 (0.5)
Neonatal brain injury	1937 (7.3)	437 (3.4)
Severe NEC^f^	546 (2.1)	91 (0.7)
Severe ROP^g^	2009 (7.6)	373 (2.9)
No BPD, neonatal brain injury, severe NEC, or severe ROP	16 069 (60.6)	10 207 (79.0)
Died between neonatal discharge and age 2 y	159 (0.6)	73 (0.6)

Abbreviations: BPD, bronchopulmonary dysplasia; NEC, necrotizing enterocolitis; ROP, retinopathy of prematurity.

^a^ Data are presented as number (percentage) of children unless otherwise indicated.

^b^ Less than 10th centile.

^c^ A complete course of steroids was defined as a course complying with the local protocol (eg, for betamethasone, two 12-mg doses given intramuscularly 24 hours apart).

^d^ As defined by Helenius et al.^28^

^e^ BPD requiring oxygen or respiratory support at 36 weeks’ postmenstrual age.

^f^ NEC confirmed at surgery.

^g^ Grade 3 or above or receiving treatment.

The proportion of children hospitalized after discharge was higher at lower gestational ages: 6138 of 10 444 children born at 31 weeks’ gestation (58.8%) had at least 1 admission, rising to 450 of 517 children born at less than 24 weeks’ gestation (87.0%) (Table 2). Most hospitalizations were nonelective (24 141 [91.1%]). The most common category of primary diagnosis for hospital admission was respiratory (*ICD-10*, chapter 10: J00-J99), with 15 754 children (40.0%) having at least 1 respiratory admission. There were 20 119 children (51.1%) who had at least 1 admission lasting 2 or more calendar days (ie, not counting shorter admissions), ranging from 4343 of 10 444 children (41.6%) born at 31 weeks’ gestation to 393 of 517 (76.3%) born at less than 24 weeks’ gestation. The median number of calendar days hospitalized between neonatal discharge and age 2 years across all admissions was 2 days (IQR, 0-7 days) and was greater for children born at a lower gestational age: median of 8 days (IQR, 3-21 days) for those born at less than 24 weeks’ gestation compared with 1 day (IQR, 0-5 days) for those born at 31 weeks’ gestation.

**Table 2.** Unadjusted Number and Percentage of Children by Gestational Age at Birth, Hospital Admissions After Neonatal Discharge Until 2 Years of Age, and Total Hospitalization Time in Calendar Days Across Admissions

Gestational age at birth, wk	Children discharged home from neonatal care, No. (%)	Children with hospital admissions between neonatal discharge and age 2 y, No./total No. (%)				Total time hospitalized across admissions, median (IQR), calendar d^a^
		Any admission	Any unplanned admission	Any respiratory admission	Any admission lasting ≥2 calendar d^a^	
<24	517 (1.3)	450/517 (87.0)	421/517 (81.4)	328/517 (63.4)	393/517 (76.3)	8 (3-21)
24	1385 (3.5)	1182/1385 (85.3)	1122/1385 (81.0)	872/1385 (63.0)	1019/1385 (73.7)	7 (2-18)
25	1979 (5.0)	1594/1979 (80.6)	1453/1979 (73.4)	1074/1979 (54.3)	1316/1979 (66.6)	5 (1-14)
26	2739 (6.9)	2130/2739 (77.8)	1970/2739 (71.9)	1431/2739 (52.2)	1753/2739 (64.0)	4 (1-11)
27	3626 (9.2)	2702/3626 (74.5)	2453/3626 (67.7)	1679/3626 (46.3)	2092/3626 (57.8)	3 (0-10)
28	4950 (12.6)	3533/4950 (71.4)	3179/4950 (64.2)	2145/4950 (43.3)	2705/4950 (54.7)	3 (0-8)
29	5908 (15.0)	3863/5908 (65.4)	3510/5908 (59.4)	2272/5908 (38.5)	2869/5908 (48.6)	2 (0-7)
30	7865 (20.0)	4906/7865 (62.4)	4426/7865 (56.3)	2738/7865 (34.8)	3629/7865 (46.2)	2 (0-6)
31	10 444 (26.5)	6138/10 444 (58.8)	5607/10 444 (53.7)	3215/10 444 (30.8)	4343/10 444 (41.6)	1 (0-5)
Total	39 413	26 498/39 413 (67.2)	24 141/39 413 (61.3)	15 754/39 413 (40.0)	20 119/39 413 (51.1)	2 (0-7)

^a^ A total of 28 children (0.1%) had incomplete data for length of hospital stay.

### Multivariable Analysis and Neonatal Morbidities

Negative binomial regression analysis showed an association between decreasing gestational age and greater total number of calendar days hospitalized across admissions (Table 3), with an AIRR for days hospitalized of 2.03 (95% CI, 1.74-2.37) for children born at less than 24 weeks’ gestation compared with those born at 31 weeks (reference group). Birth at 30 weeks’ (vs 31 weeks’) gestation was associated with a smaller increase in days hospitalized (AIRR, 1.08; 95% CI, 1.03-1.13). SGA was associated with an increased AIRR (1.39; 95% CI, 1.31-1.48), as was neonatal discharge in the spring (AIRR, 1.08; 95% CI, 1.03-1.13), fall (AIRR, 1.18; 95% CI, 1.12-1.23), and winter (AIRR, 1.17; 95% CI, 1.11-1.22) compared with summer. Examining neonatal morbidities (Table 3), the greatest increases in hospitalization days were associated with presence vs absence of BPD (AIRR, 1.80; 95% CI, 1.72-1.88) and severe NEC (AIRR, 1.88; 95% CI, 1.65-2.15), with a somewhat smaller increase for neonatal brain injury (AIRR, 1.46; 95% CI, 1.36-1.57) and smaller still for severe ROP (AIRR, 1.20; 95% CI, 1.11-1.30).

**Table 3.** Negative Binomial Regression Model for Associations Between Child Characteristics and Total Calendar Days Hospitalized Across Admissions Between Neonatal Discharge and Age 2 Years^a^

Variable	AIRR (95% CI)	*P* value^b^
Gestational age at birth, wk		
<24	2.03 (1.74-2.37)	<.001
24	1.75 (1.63-2.01)	<.001
25	1.47 (1.34-1.60)	<.001
26	1.43 (1.33-1.54)	<.001
27	1.32 (1.22-1.40)	<.001
28	1.24 (1.17-1.32)	<.001
29	1.17 (1.10-1.23)	<.001
30	1.08 (1.03-1.13)	<.001
31	1 [Reference]	NA
Sex		
Female	1 [Reference]	NA
Male	0.86 (0.83-0.89)	<.001
Small for gestational age	1.39 (1.31-1.48)	<.001
Season of neonatal discharge		
Spring (March-May)	1.08 (1.03-1.13)	<.001
Summer (June-August)	1 [Reference]	NA
Fall (September-November)	1.18 (1.12-1.23)	<.001
Winter (December-February)	1.17 (1.11-1.22)	<.001
Neonatal morbidities, presence vs absence		
BPD^c^	1.80 (1.72-1.88)	<.001
Severe NEC^d^	1.88 (1.65-2.15)	<.001
Neonatal brain injury	1.46 (1.36-1.57)	<.001
Severe ROP^e^	1.20 (1.11-1.30)	<.001

Abbreviations: AIRR, adjusted incidence rate ratio; BPD, bronchopulmonary dysplasia; NA, not applicable; NEC, necrotizing enterocolitis; ROP, retinopathy of prematurity.

^a^ N = 39 002 after exclusions for missing data.

^b^ *P* value for likelihood ratio test of α = 0 was <.001.

^c^ Requiring oxygen or respiratory support at 36 weeks’ postmenstrual age.

^d^ Confirmed at surgery.

^e^ Grade 3 or above or receiving treatment.

Subgroup analyses by gestational age groups (due to varying prevalence of morbidities) showed that neonatal morbidities were associated with a greater increase in hospitalization days for children born at 28 through 31 weeks’ gestation compared with children born at less than 28 weeks’ gestation; for example, the AIRR for children with BPD born at 28 through 31 weeks’ gestation was 1.96 (95% CI, 1.85-2.08), compared with 1.53 (95% CI, 1.43-1.63) for those born at less than 28 weeks (eTable 1 in Supplement 1). An association between days hospitalized and being discharged in fall or winter (compared with summer) was only present among children born at 28 through 31 weeks’ gestation (fall: AIRR, 1.25 [95% CI, 1.18-1.33] ; winter: AIRR, 1.20 [95% CI, 1.13-1.27]) and not those born at less than 28 weeks (fall: AIRR, 1.00 [95% CI, 0.92-1.08]; winter: AIRR, 1.09 [95% CI, 1.00-1.18]).

**Figure 2.**
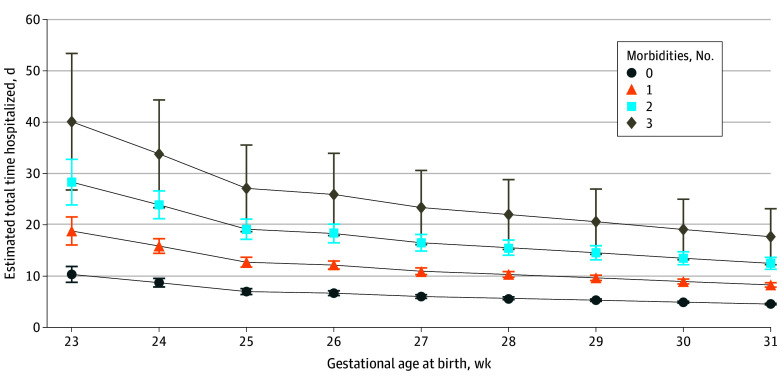
Estimates of Expected Number of Total Calendar Days of Hospitalization After Neonatal Discharge Until 2 Years of Age, by Gestational Age and Count of Neonatal Morbidities Whiskers represent 95% CIs. Neonatal morbidities included bronchopulmonary dysplasia, severe necrotizing enterocolitis, and neonatal brain injury.

We used the model to estimate total expected number of calendar days hospitalized across all admissions after neonatal discharge by gestational age at birth and the neonatal morbidities of BPD, severe NEC, and brain injury (the morbidities with the greatest increase in AIRR), with other variables held at mean values (eFigure in Supplement 1). A further model was developed using the count of these morbidities (from 0-3) (eTable 2 in Supplement 1). Outputs from this model produced estimates of total expected days hospitalized (Figure 2); for example, we estimated that a child born at less than 24 weeks’ gestation with a single morbidity (eg, BPD) would have 18.3 (95% CI, 15.1-29.0) total days hospitalized before 2 years of age, rising to 40.6 (95% CI, 34.8-44.3) days for the same child if they had 3 morbidities. For a child born at 31 weeks’ gestation, the estimate rose from 4.6 (95% CI, 3.7-7.1) days for no morbidities to 13.3 (95% CI, 10.8-19.4) days for 2 morbidities—although the number of actual children born at 31 weeks’ gestation who have such morbidity is small, and the estimates had wide 95% CIs. The *P* values for the likelihood ratio test of α = 0 for overdispersion were <.001, confirming appropriateness of negative binomial rather than Poisson regression, as the former is able to deal with data that have a high degree of variance (ie, some children with long hospitalizations) that do not meet the assumptions for Poisson regression.

When we examined the primary-diagnosis *ICD-10* codes across all admissions between neonatal discharge and the age of 2 years, the largest groups were respiratory infections (J20-J22 [“other acute lower respiratory infections”]: 22 508 of the 92 305 hospitalization spells with admission diagnosis data [24.4%] and J00-J06 [“acute upper respiratory infections”]: 9492 spells [10.3%]) (eTable 3 in Supplement 1). To account for differences in length of neonatal hospitalization, we also performed sensitivity analyses excluding children with total stays of more than 80 days (>99th centile; n = 391 of 39 385 children with hospitalization data at discharge from neonatal care [1.0%]) to reduce influence of unusually long stays (eTable 4 in Supplement 1), analyses using total days hospitalized for nonelective admissions only (eTable 5 in Supplement 1), and analyses including only hospitalizations 1 year after neonatal discharge (eTable 6 in Supplement 1). Results were similar, with the same directions of associations across analyses.

In addition, we examined the largest group of admissions, those with primary respiratory diagnoses (eTable 7 in Supplement 1). There were 15 754 children with any respiratory admission (40.0%). For these admissions, number of days hospitalized was associated with BPD (AIRR, 2.10; 95% CI, 1.98-2.24) and neonatal brain injury (AIRR, 1.32; 95% CI, 1.19-1.46) but not severe NEC (AIRR, 1.07; 95% CI, 0.88-1.30) after adjustment for other variables, unlike in the primary analysis. However, even among children with severe NEC or neonatal brain injury, there were greater numbers of admissions for respiratory conditions than for specific complications of other conditions (eTable 3 in Supplement 1).

## Discussion

Using contemporary national population-level linked data, we quantified hospital use up to the age of 2 years among children born very preterm by both gestational age and morbidity. We found that most children born before 32 weeks’ gestation had at least 1 episode of hospital admission between neonatal discharge and their second birthday. Although across the whole cohort the median number of calendar days hospitalized was only 2 (and 67.2% of children had at least 1 admission), the most preterm-born children (born before 24 weeks’ gestation) had a median of 8 days hospitalized, and 87.0% had at least 1 admission. Most children in the cohort did not have major neonatal morbidity; however, the presence of increasing numbers of morbidities was associated with greater numbers of total days hospitalized across admissions, and the model estimated that the most preterm-born children with 3 major morbidities may have up to 40.6 days of hospitalization. Morbidities were particularly important for children born at 28 through 31 weeks’ gestation, increasing the duration of hospitalization considerably more than for their peers of similar gestational age without the same morbidities. Regardless of the nature of morbidities, children were commonly hospitalized for respiratory infections, with smaller numbers of admissions associated with other morbidities, such as for stoma complications following severe NEC.

Comparing our results with previous literature, a cohort study of births in England from 2005 to 2006 found that 94% of children born at less than 28 weeks’ gestation were admitted at least once between neonatal discharge and 10 years of age (a longer period of follow-up than in our study); this percentage was 82% for children born at 30 to 31 weeks’ gestation and 51% for those born at 40 weeks.^29^ Examining international literature, a US study found a lower percentage of preterm-born children with hospital admissions than observed from UK data: for children born at less than 26 weeks’ gestation in the US study, one-quarter were hospitalized before 1 year of age, most commonly for respiratory conditions (50%) or surgery (26%).^30^ Other studies concur with our findings that respiratory conditions were the most common cause of hospitalization among preterm-born children.^31,32^ Despite advances in care, complications of preterm birth are not decreasing,^3,4,5^ which may be reflected in considerable numbers of children requiring hospitalization. Another US study, from the 1990s, found children born at 31 weeks’ gestation spent 8 days hospitalized, on average, between neonatal discharge and 1 year of age, increasing to 12 days for those born at less than 25 weeks’ gestation^33^—longer than observed within our data. There are differences in service delivery and access to pediatric care between the UK and the US. Children in the UK may access short-stay pediatric assessment units (classified as hospital admissions^34^), whereas in a US setting, similar children may attend a same-day pediatric office appointment (something not routinely available within the UK); we observed that many children in our study had short (1 calendar day) admissions, and this may explain the lower percentage of preterm-born children hospitalized in the US studies.

Comparing admission rates with children born at 37 weeks’ gestation or later, a large study of births from 2001 to 2011 in New South Wales, Australia, found that 16.8% of children born at 37 or more weeks’ gestation had at least 1 hospital admission before the age of 1 year, whereas 62.1% of children born at 24 to 27 weeks’ gestation and 47.7% of those born at 28 to 31 weeks’ gestation had at least 1 hospital admission before 1 year of age.^6^ It is clear that preterm-born children have greater use of hospital services than those born at 37 weeks’ gestation or later. While the association between lower gestational age and hospitalization has been observed previously,^6^ we are not aware of previous studies that quantify other associations found in the current study, such as between neonatal morbidities and hospitalizations, using large population-level data.

In this study, respiratory infections accounted for the most hospital admissions, even for children whose ongoing morbidity was in the neurological and/or gastrointestinal system; moreover, fall and winter neonatal discharges were associated with increased subsequent hospitalization, consistent with previous research.^24^ High-income countries are commencing maternal or infant immunization programs for respiratory syncytial virus (RSV),^35,36^ and early evidence suggests this may reduce hospitalizations for RSV bronchiolitis at a population level.^36,37^ However, it is vital for all countries to monitor hospital admissions, especially in high-risk populations such as children born very preterm, to determine whether vaccination strategies are effective and whether health care provision is sufficient for the increasing number of children with morbidity from the neonatal period.^3,4,5^

Other measures to prevent hospitalization could include additional follow-up and outreach services; however, this requires further investigation. Our study findings may also aid discussions with families before neonatal discharge, which could include general health advice, such as handwashing and accessing health care for assessment of respiratory infections.^38^

Preterm birth is a major factor associated with medical complexity in children,^39^ and in this group of children the impact of hospitalization is considerable.^9,12^ This study found that major neonatal morbidity was associated with significant consequences for children born very preterm and their families in terms of more time spent in hospital, which adds to findings of previous studies demonstrating the association between the count of neonatal morbidities and death and neurodisability in childhood.^13,21,40,41^ Taken together, there is growing evidence that the number of morbidities may be an important neonatal outcome within epidemiologic studies or clinical trials. Given the ability to assess for this at the point of neonatal discharge and the ability to rank the number of morbidities, this may be considered for use as a trial end point as an ordinal scale from 0 morbidities through to death using the Desirability of Outcome Ranking method.^14,42^ This could be of particular relevance for future neonatal platform trials, which could be adapted without waiting for 2-year outcomes.^43^

### Limitations

This study has limitations. Although it used a novel dataset linkage to provide population-level adjusted analyses using reliable and detailed neonatal data, analysis was based on linkage of routinely collected data, with the associated risks of failed linkage, missing data, and misclassification despite data validation and cleaning. We found that 3.1% of children in the study dataset had failed linkages, though it is not possible to quantify other potential missing admissions. The percentage of missing data was low across variables and outcomes (Table 1); therefore, we were able to use complete case analysis. To reduce identifiable information, we were limited in the granularity of dates of discharges and admissions; therefore, we used admission source and method variables with the aim of avoiding misclassification of hospitalizations, although the risk of erroneous data remained. In addition, recording of morbidities may be incomplete, although data in the NNRD remain comparable to trial data.^44^

While combining different neonatal morbidities into a single count provides a useful outcome, it simplifies morbidities, and we lacked data for severity of morbidities (eg, BPD grade). Selection of morbidities across studies and datasets may vary, and not all morbidities will be included; for example, we did not include late-onset sepsis, as it was incompletely validated during the period of NNRD data collection (2013-2018).^45^ The prevalence or AIRRs for hospitalization were not identical between morbidities, and AIRRs differed between subgroups. Due to the comparatively small number of children with 3 morbidities, the 95% CIs for estimates of expected hospitalization days were wide, and it may be difficult to draw conclusions that are generalizable, especially at later gestational ages. The use of the neonatal morbidity count may need adjustment for the morbidities used, depending on the population examined, severity of morbidity, size of the study, and ability to collect data. We examined associations at a population level; however, there are unmeasured confounders for which we did not account (eg, home environment), and further research would be required to make individual-level predictions.

Significant associations should be interpreted in the context that numerous variables were assessed, increasing the probability of a type I error; however, the *P* values observed for important associations were generally <.001. In addition, due to differences between pediatric services within the UK compared with health care systems in other high-income countries, there may be limitations in the generalizability of this study internationally.

## Conclusions

In this cohort study using nationwide data in England and Wales, most children born before 32 weeks’ gestation required hospitalization after neonatal discharge and before the age of 2 years. Greater total duration of hospitalization was associated with lower gestational age and the number of major neonatal morbidities developed during the neonatal stay. This information may assist discussions with families around the time of neonatal discharge, assist with the development and provision of services for these children, and provide a baseline from which to investigate interventions to reduce hospitalization. In addition, the number of major neonatal morbidities was associated with the total days of hospitalization, a relevant outcome for families, and could be considered as a useful surrogate outcome in neonatal trials.

## Notes

Supplement 1. **eTable 1.** Negative Binomial Regression Model for Total Days Hospitalized (in Calendar Days) Between Neonatal Discharge and the Age of 2 Years, Subgroup Analysis **eTable 2.** Negative Binomial Regression Model for Total Days Hospitalized (in Calendar Days) Between Neonatal Discharge and the Age of 2 Years, Adjusting for Gestation and Count of Neonatal Morbidities (n = 39 002) **eTable 3.** Ten Most Frequent Primary Admission *ICD-10* Diagnoses Across all Hospitalizations After Neonatal Discharge and Until 2 Years of Age, for All Children and Then by Neonatal Morbidity **eTable 4.** Negative Binomial Regression Model for Total Days Hospitalized (in Calendar Days) Between Neonatal Discharge and the Age of 2 Years, Excluding Children With Total Stays >99th Centile (>80 days) (n = 38 624) **eTable 5.** Negative Binomial Regression Model for Total Days Hospitalized (in Calendar Days) for Nonelective Admissions Between Neonatal Discharge and the Age of 2 Years (n = 39 002) **eTable 6.** Negative Binomial Regression Model for Total Days Hospitalized (in Calendar Days) 1 Chronological Year After Neonatal Discharge (n = 39 002) **eTable 7.** Negative Binomial Regression Model for Total Days Hospitalized (in Calendar Days) With Respiratory Primary Diagnoses Between Neonatal Discharge and the Age of 2 Years (n = 39 002) **eFigure.** Estimated Number of Calendar Days Hospitalized After Neonatal Discharge and Until 2 Years of Age, by Gestation and Neonatal Morbidities

Supplement 2.
UK Neonatal Collaborative

Supplement 3.
Data Sharing Statement
