# Supplementary material for: Neonatal Morbidities and Hospitalization in the First 2 Years of Life Among Infants Born Very Preterm
Source: JAMA Netw Open. 2025 Sep 3;8(9):e2530123. doi: 10.1001/jamanetworkopen.2025.30123 (PMC12409584; doi:10.1001/jamanetworkopen.2025.30123)
Supplement: Supplement 1. — eTable 1. Negative Binomial Regression Model for Total Days Hospitalized (in Calendar Days) Between Neonatal Discharge and the Age of 2 Years, Subgroup Analysis eTable 2. Negative Binomial Regression Model for Total Days Hospitalized (in Calendar Days) Between Neonatal Discharge and the Age of 2 Years, Adjusting for Gestation and Count of Neonatal Morbidities (n = 39 002) eTable 3. Ten Most Frequent Primary Admission ICD-10 Diagnoses Across all Hospitalizations After Neonatal Discharge and Until 2 Years of Age, for All Children and Then by Neonatal Morbidity eTable 4. Negative Binomial Regression Model for Total Days Hospitalized (in Calendar Days) Between Neonatal Discharge and the Age of 2 Years, Excluding Children With Total Stays >99th Centile (>80 days) (n = 38 624) eTable 5. Negative Binomial Regression Model for Total Days Hospitalized (in Calendar Days) for Nonelective Admissions Between Neonatal Discharge and the Age of 2 Years (n = 39 002) eTable 6. Negative Binomial Regression Model for Total Days Hospitalized (in Calendar Days) 1 Chronological Year After Neonatal Discharge (n = 39 002) eTable 7. Negative Binomial Regression Model for Total Days Hospitalized (in Calendar Days) With Respiratory Primary Diagnoses Between Neonatal Discharge and the Age of 2 Years (n = 39 002) eFigure. Estimated Number of Calendar Days Hospitalized After Neonatal Discharge and Until 2 Years of Age, by Gestation and Neonatal Morbidities [file jamanetwopen-e2530123-s001.pdf]

Supplementary Online Content

van Hasselt TJ, Dorner RA, Katheria A, et al; United Kingdom Neonatal Collaborative. Neonatal morbidities and hospitalization in the first 2 years of life among infants born very preterm. *JAMA Netw Open*. 2025;8(9):e2530123. doi:10.1001/jamanetworkopen.2025.30123

eTable 1. Negative Binomial Regression Model for Total Days Hospitalized (in Calendar Days) Between Neonatal Discharge and the Age of 2 Years, Subgroup Analysis

eTable 2. Negative Binomial Regression Model for Total Days Hospitalized (in Calendar Days) Between Neonatal Discharge and the Age of 2 Years, Adjusting for Gestation and Count of Neonatal Morbidities (n = 39 002)

eTable 3. Ten Most Frequent Primary Admission *ICD-10* Diagnoses Across all Hospitalizations After Neonatal Discharge and Until 2 Years of Age, for All Children and Then by Neonatal Morbidity

eTable 4. Negative Binomial Regression Model for Total Days Hospitalized (in Calendar Days) Between Neonatal Discharge and the Age of 2 Years, Excluding Children With Total Stays >99th Centile (>80 days) (n = 38 624)

eTable 5. Negative Binomial Regression Model for Total Days Hospitalized (in Calendar Days) for Nonelective Admissions Between Neonatal Discharge and the Age of 2 Years (n = 39 002)

eTable 6. Negative Binomial Regression Model for Total Days Hospitalized (in Calendar Days) 1 Chronological Year After Neonatal Discharge (n = 39 002)

eTable 7. Negative Binomial Regression Model for Total Days Hospitalized (in Calendar Days) With Respiratory Primary Diagnoses Between Neonatal Discharge and the Age of 2 Years (n = 39 002)

eFigure. Estimated Number of Calendar Days Hospitalized After Neonatal Discharge and Until 2 Years of Age, by Gestation and Neonatal Morbidities

This supplementary material has been provided by the authors to give readers additional information about their work.

© 2025 van Hasselt TJ et al. *JAMA Network Open*.

eTable 1 – Negative binomial regression model for total days hospitalized (in calendar days) between neonatal discharge and the age of 2 years, subgroup analysis

Variables	Subgroup analysis <28 weeks (n=10,152)		Subgroup analysis 28-31 weeks (n=28,850)	
	Adjusted incident rate ratio (95% confidence interval)	*p*	Adjusted incident rate ratio (95% confidence interval)	*p*
**Gestation at birth (weeks)**			-	
**<24**	1.66 (1.44 to 1.91)	<0.001	-	
**24**	1.42 (1.29 to 1.57)	<0.001	-	
**25**	1.18 (1.08 to 1.28)	<0.001	-	
**26**	1.13 (1.05 to 1.22)	<0.001	-	
**27**	1 (Reference)	0.001	-	
**28**	-		1.21 (1.14 to 1.29)	<0.001
**29**	-		1.15 (1.09 to 1.21)	<0.001
**30**	-		1.07 (1.02 to 1.13)	0.009
**31**	-		1 (Reference)	-
**Sex**				
**Male**	0.89 (0.84 to 0.94)	<0.001	0.85 (0.81 to 0.88)	<0.001
**Female**	1 (Reference)	-	1 (Reference)	-
**Small for gestational age**				
**Present**	1.32 (1.15 to 1.51)	<0.001	1.38 (1.29 to 1.49)	<0.001
**Season of neonatal discharge**				
**Spring (March-May)**	0.92 (0.85 to 1.00)	0.055	1.14 (1.08 to 1.21)	<0.001
**Summer (June-August)**	1 (Reference)	-	1 (Reference)	-
**Fall (September-November)**	1.00 (0.92 to 1.08)	0.960	1.25 (1.18 to 1.33)	<0.001
**Winter (December-February)**	1.09 (1.00 to 1.18)	0.048	1.20 (1.13 to 1.27)	<0.001
**Neonatal morbidities**				
**BPD**	1.53 (1.43 to 1.63)	<0.001	1.96 (1.85 to 2.08)	<0.001
**Severe NEC**	1.74 (1.51 to 2.00)	<0.001	2.29 (1.77 to 2.96)	<0.001
**Brain injury**	1.25 (1.15 to 1.35)	<0.001	1.78 (1.58 to 2.00)	<0.001
**Severe ROP**	1.28 (1.18 to 1.38)	<0.001	1.04 (0.87 to 1.24)	0.689

p-value for likelihood ratio test of alpha=0 <0.001 for both analyses

© 2025 van Hasselt TJ et al. *JAMA Network Open*.

eTable 2 – Negative binomial regression model for total days hospitalized (in calendar days) between neonatal discharge and the age of 2 years, adjusting for gestation and count of neonatal morbidities (n= 39,002)

Variables	Adjusted incident rate ratio (95% confidence interval)	*p*
**Gestation at birth (weeks)**		
**<24**	2.27 (1.95 to 2.64)	<0.001
**24**	1.91 (1.73 to 2.12)	<0.001
**25**	1.53 (1.41 to 1.67)	<0.001
**26**	1.47 (1.36 to 1.58)	<0.001
**27**	1.32 (1.24 to 1.41)	<0.001
**28**	1.24 (1.17 to 1.32)	<0.001
**29**	1.17 (1.10 to 1.23)	<0.001
**30**	1.08 (1.03 to 1.13)	0.003
**31**	1 (Reference)	-
**Sex**		
**Male**	0.86 (0.83 to 0.88)	<0.001
**Female**	1 (Reference)	-
**Small for gestational age**		
**Present**	1.41 (1.32 to 1.50)	<0.001
**Season of neonatal discharge**		
**Spring (March-May)**	1.08 (1.03 to 1.14)	0.001
**Summer (June-August)**	1 (Reference)	-
**Fall (September-November)**	1.18 (1.13 to 1.24)	<0.001
**Winter (December-February)**	1.17 (1.12 to 1.23)	<0.001
**Sum of neonatal morbidities**		
**0**	1 (Reference)	-
**1**	1.82 (1.74 to 1.90)	<0.001
**2**	2.74 (2.51 to 2.99)	<0.001
**3**	3.88 (2.85 to 5.27)	<0.001

p-value for likelihood ratio test of alpha=0 <0.001

© 2025 van Hasselt TJ et al. *JAMA Network Open*.

eTable 3 – Ten most frequent primary admission ICD10 diagnoses across all hospitalizations after neonatal discharge and until 2 years of age, for all children and then by neonatal morbidity

ICD10 Code	Frequency	Percent
**All neonatal discharges**		
J20-J22 Other acute lower respiratory infections	22,508	24.4
J00-J06 Acute upper respiratory infections	9,492	10.3
B349 Viral infection, unspecified	8,406	9.1
R50-R69 General symptoms and signs	4,398	4.8
R00-R09 Symptoms and signs involving the circulatory and respiratory systems	3,919	4.3
K40-K46 Hernia	3,821	4.1
K20-K31 Diseases of oesophagus, stomach and duodenum	2,687	2.9
Z00-Z13 Persons encountering health services for examination and investigation	2,255	2.4
P20-P29 Respiratory and cardiovascular disorders specific to the perinatal period	2,111	2.3
J09-J18 Influenza and pneumonia	1,988	2.2
**Children with BPD**		
J20-J22 Other acute lower respiratory infections	10,554	25.6
J00-J06 Acute upper respiratory infections	4,349	10.5
B349 Viral infection, unspecified	3,570	8.7
R00-R09 Symptoms and signs involving the circulatory and respiratory systems	1,785	4.3
R50-R69 General symptoms and signs	1,778	4.3
P20-P29 Respiratory and cardiovascular disorders specific to the perinatal period	1,655	4.0
K40-K46 Hernia	1,492	3.6
J09-J18 Influenza and pneumonia	1,034	2.5
K20-K31 Diseases of oesophagus, stomach and duodenum	1,028	2.5
Z00-Z13 Persons encountering health services for examination and investigation	946	2.3
**Children with NEC**		
J20-J22 Other acute lower respiratory infections	558	19.3
J00-J06 Acute upper respiratory infections	213	7.4
B349 Viral infection, unspecified	197	6.8
Z40-Z54 Persons encountering health services for specific procedures and health care	139	4.8
R50-R69 General symptoms and signs	125	4.3
R10-R19 Symptoms and signs involving the digestive system and abdomen	101	3.5
R00-R09 Symptoms and signs involving the circulatory and respiratory systems	93	3.2
K55-K64 Other diseases of intestines	89	3.1
K90-K93 Other diseases of the digestive system	88	3.1
Z00-Z13 Persons encountering health services for examination and investigation	81	2.8
**Children with brain injury**		
J20-J22 Other acute lower respiratory infections	2,047	20.6
J00-J06 Acute upper respiratory infections	994	10.0
B349 Viral infection, unspecified	737	7.4
R50-R69 General symptoms and signs	565	5.7
R00-R09 Symptoms and signs involving the circulatory and respiratory systems	384	3.9
P20-P29 Respiratory and cardiovascular disorders specific to the perinatal period	295	3.0
T80-T88 Complications of surgical and medical care, not elsewhere classified	285	2.9
K40-K46 Hernia	278	2.8
K20-K31 Diseases of oesophagus, stomach and duodenum	272	2.7
Z00-Z13 Persons encountering health services for examination and investigation	251	2.5

© 2025 van Hasselt TJ et al. *JAMA Network Open*.

eTable 4 – Negative binomial regression model for total days hospitalized (in calendar days) between neonatal discharge and the age of 2 years, excluding children with total stays >99th centile (>80 days) (n= 38,624)

Variables	Adjusted incident rate ratio (95% confidence interval)	p
**Gestation at birth (weeks)**		
**<24**	1.72 (1.48 to 2.00)	<0.001
**24**	1.74 (1.58 to 1.93)	<0.001
**25**	1.57 (1.44 to 1.71)	<0.001
**26**	1.49 (1.38 to 1.60)	<0.001
**27**	1.35 (1.26 to 1.43)	<0.001
**28**	1.28 (1.21 to 1.35)	<0.001
**29**	1.16 (1.11 to 1.23)	<0.001
**30**	1.17 (1.11 to 1.22)	<0.001
**31**	1 (Reference)	-
**Sex**		
**Male**	0.81 (0.79 to 0.84)	
**Female**	1 (Reference)	-
**Small for gestational age**		
**Present**	1.20 (1.13 to 1.27)	<0.001
**Season of neonatal discharge**		
**Spring (March-May)**	1.01 (0.96 to 1.05)	0.800
**Summer (June-August)**	1 (Reference)	-
**Fall (September-November)**	1.13 (1.08 to 1.18)	<0.001
**Winter (December-February)**	1.09 (1.04 to 1.14)	<0.001
**Neonatal morbidities**		
**BPD**	1.59 (1.53 to 1.66)	<0.001
**Severe NEC**	1.70 (1.50 to 1.93)	<0.001
**Brain injury**	1.35 (1.26 to 1.44)	<0.001
**Severe ROP**	1.17 (1.09 to 1.26)	<0.001

p-value for likelihood ratio test of alpha=0 <0.001

© 2025 van Hasselt TJ et al. *JAMA Network Open*.

eTable 5 – Negative binomial regression model for total days hospitalized (in calendar days) for non-elective admissions between neonatal discharge and the age of 2 years (n= 39,002)

Variables	Adjusted incident rate ratio (95% confidence interval)	*p*
**Gestation at birth (weeks)**		
**<24**	2.02 (1.71 to 2.39)	<0.001
**24**	1.80 (1.60 to 2.01)	<0.001
**25**	1.45 (1.32 to 1.59)	<0.001
**26**	1.37 (1.26 to 1.48)	<0.001
**27**	1.28 (1.19 to 1.38)	<0.001
**28**	1.22 (1.14 to 1.30)	<0.001
**29**	1.14 (1.08 to 1.21)	<0.001
**30**	1.06 (1.01 to 1.12)	0.028
**31**	1 (Reference)	-
**Sex**		
**Male**	0.88 (0.85 to 0.92)	<0.001
**Female**	1 (Reference)	-
**Small for gestational age**		
**Present**	1.35 (1.26 to 1.44)	<0.001
**Season of neonatal discharge**		
**Spring (March-May)**	1.08 (1.03 to 1.14)	0.002
**Summer (June-August)**	1 (Reference)	-
**Fall (September-November)**	1.20 (1.14 to 1.27)	<0.001
**Winter (December-February)**	1.17 (1.11 to 1.23)	<0.001
**Neonatal morbidities**		
**BPD**	1.78 (1.70 to 1.87)	<0.001
**Severe NEC**	1.70 (1.47 to 1.96)	<0.001
**Brain injury**	1.42 (1.31 to 1.53)	<0.001
**Severe ROP**	1.18 (1.09 to 1.28)	<0.001

p-value for likelihood ratio test of alpha=0 <0.001

© 2025 van Hasselt TJ et al. *JAMA Network Open*.

eTable 6 – Negative binomial regression model for total days hospitalized (in calendar days) one chronological year after neonatal discharge (n= 39,002)

Variables	Adjusted incident rate ratio (95% confidence interval)	*p*
**Gestation at birth (weeks)**		
**<24**	2.23 (1.88 to 2.65)	<0.001
**24**	1.85 (1.65 to 2.08)	<0.001
**25**	1.58 (1.43 to 1.74)	<0.001
**26**	1.47 (1.35 to 1.60)	<0.001
**27**	1.33 (1.24 to 1.43)	<0.001
**28**	1.25 (1.18 to 1.34)	<0.001
**29**	1.16 (1.10 to 1.24)	<0.001
**30**	1.07 (1.01 to 1.13)	0.013
**31**	1 (Reference)	-
**Sex**		
**Male**	0.84 (0.81 to 0.87)	<0.001
**Female**	1 (Reference)	-
**Small for gestational age**		
**Present**	1.51	
**Season of neonatal discharge**		
**Spring (March-May)**	1.12 (1.06 to 1.18)	<0.001
**Summer (June-August)**	1 (Reference)	-
**Fall (September-November)**	1.23 (1.17 to 1.29)	<0.001
**Winter (December-February)**	1.22 (1.16 to 1.28)	<0.001
**Neonatal morbidities**		
**BPD**	1.79 (1.71 to 1.88)	<0.001
**Severe NEC**	2.01 (1.74 to 2.32)	<0.001
**Brain injury**	1.41 (1.30 to 1.52)	<0.001
**Severe ROP**	1.18 (1.08 to 1.28)	<0.001

p-value for likelihood ratio test of alpha=0 <0.001

© 2025 van Hasselt TJ et al. *JAMA Network Open*.

eTable 7 – Negative binomial regression model for total days hospitalized (in calendar days) with respiratory primary diagnoses between neonatal discharge and the age of 2 years (n= 39,002)

Variables	Adjusted incident rate ratio (95% confidence interval)	*p*
**Gestation at birth (weeks)**		
**<24**	2.58 (2.07 to 3.23)	<0.001
**24**	2.28 (1.96 to 2.65)	<0.001
**25**	1.70 (1.50 to 1.93)	<0.001
**26**	1.56 (1.40 to 1.74)	<0.001
**27**	1.49 (1.36 to 1.64)	<0.001
**28**	1.38 (1.27 to 1.50)	<0.001
**29**	1.29 (1.20 to 1.40)	<0.001
**30**	1.12 (1.04 to 1.20)	0.002
**31**	1 (Reference)	-
**Sex**		
**Male**	0.93 (0.89 to 0.98)	0.003
**Female**	1 (Reference)	-
**Small for gestational age**		
**Present**	1.30 (1.19 to 1.42)	<0.001
**Season of neonatal discharge**		
**Spring (March-May)**	1.05 (0.98 to 1.12)	0.186
**Summer (June-August)**	1 (Reference)	-
**Fall (September-November)**	1.44 (1.34 to 1.54)	<0.001
**Winter (December-February)**	1.22 (1.14 to 1.30)	<0.001
**Neonatal morbidities**		
**BPD**	2.10 (1.98 to 2.24)	<0.001
**Severe NEC**	1.07 (0.88 to 1.30)	0.481
**Brain injury**	1.32 (1.19 to 1.46)	<0.001
**Severe ROP**	1.11 (1.00 to 1.25)	0.061

p-value for likelihood ratio test of alpha=0 <0.001

© 2025 van Hasselt TJ et al. *JAMA Network Open*.

eFigure – Estimated number of calendar days hospitalized after neonatal discharge and until 2 years of age, by gestation and neonatal morbidities

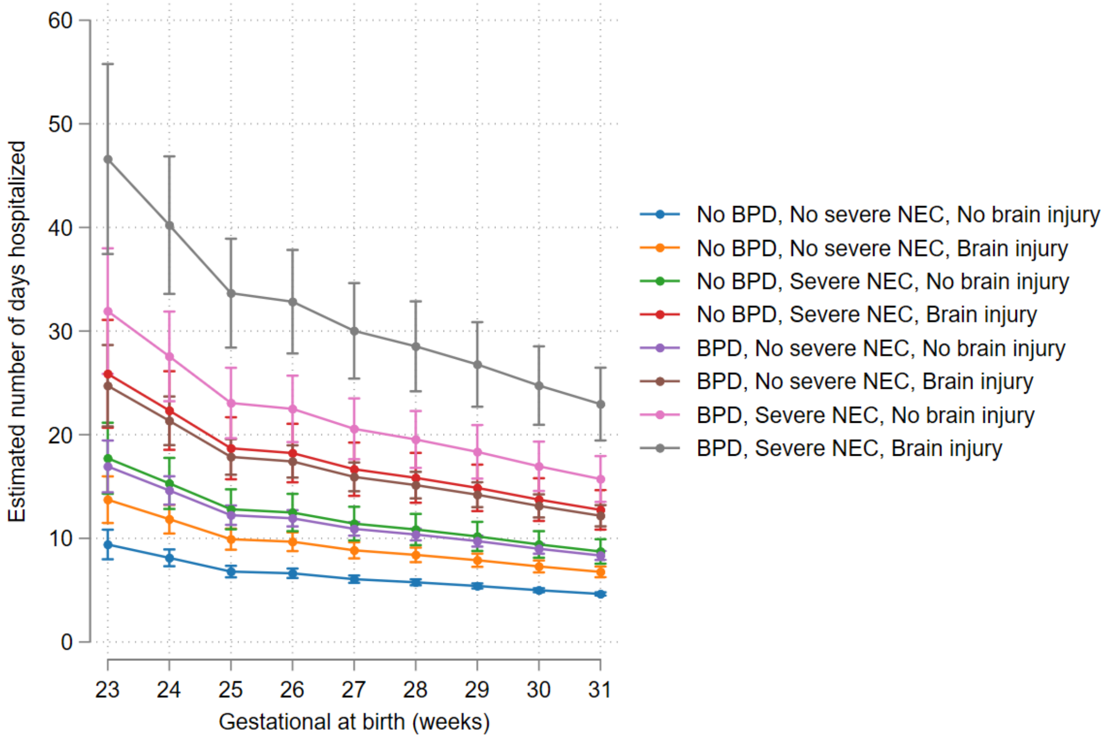

© 2025 van Hasselt TJ et al. *JAMA Network Open*.
