# Supplementary material for: Neonatal Morbidities and Hospitalization in the First 2 Years of Life Among Infants Born Very Preterm
Source: JAMA Netw Open. 2025 Sep 3;8(9):e2530123. doi: 10.1001/jamanetworkopen.2025.30123 (PMC12409584; doi:10.1001/jamanetworkopen.2025.30123)
Supplement: Supplement 2. — UK Neonatal Collaborative [file jamanetwopen-e2530123-s002.pdf]

Supplemental Online Content: Nonauthor Collaborators
*First name, last name, and suffix (if applicable) are required and will appear in PubMed.

*Group Name(s): UK Neonatal Collaborative							
***First Name and Middle Initial(s)**	***Last Name**	***Suffix (eg, Jr, III)**	Academic Degrees	Institution	Location (city, state/province, country)	Role or Contribution, eg, chair, principal investigator	Group (if more than 1 Group listed in the byline) and/or Subgroup (eg, Steering Committee)
Matthew	Babirecki			Airedale General Hospital	England	UKNC Lead	
Rebecca	Kettle			Alder Hey	England	UKNC Lead	
Anand	Kamalanathan			Arrowe Park Hospital	England	UKNC Lead	
Clare	Cane			Barnet Hospital	England	UKNC Lead	
Kavi	Aucharaz			Barnsley District General Hospital	England	UKNC Lead	
Rathod	Poorva			Basildon Hospital	England	UKNC Lead	
Jummy	Awoseyila			Basingstoke & North Hampshire Hospital	England	UKNC Lead	
L M	Wong			Bassetlaw District General Hospital	England	UKNC Lead	
Anita	Mittal			Bedford Hospital	England	UKNC Lead	
Penny	Broggio			Birmingham City Hospital	England	UKNC Lead	
Pinki	Surana			Birmingham Heartlands Hospital	England	UKNC Lead	
Matt	Nash			Birmingham Women's Hospital	England	UKNC Lead	
Sam	Sam Wallis			Bradford Royal Infirmary	England	UKNC Lead	
Ahmed	Hassan			Broomfield Hospital, Chelmsford	England	UKNC Lead	
Karin	Schwarz			Calderdale Royal Hospital	England	UKNC Lead	
Shu-Ling	Chuang			Chelsea & Westminster Hospital	England	UKNC Lead	
Penelope	Young			Chesterfield & North Derbyshire Royal Hospital	England	UKNC Lead	
Romona	Onita			Colchester General Hospital	England	UKNC Lead	
Mani	Kandasamy			Conquest Hospital	England	UKNC Lead	
Stephen	Brearey			Countess of Chester Hospital	England	UKNC Lead	
Joselyn	Morris			Croydon University Hospital	England	UKNC Lead	

Supplemental Online Content: Nonauthor Collaborators
*First name, last name, and suffix (if applicable) are required and will appear in PubMed.

*First Name and Middle Initial(s)	*Last Name	*Suffix (eg, Jr, III)	Academic Degrees	Institution	Location (city, state/province, country)	Role or Contribution, eg, chair, principal investigator	Group (if more than 1 Group listed in the byline) and/or Subgroup (eg, Steering Committee)
Rachel	Smith			Cumberland Infirmary	England	UKNC Lead	
Bharath	Gowda			Darent Valley Hospital	England	UKNC Lead	
Mehdi	Garbash			Darlington Memorial Hospital	England	UKNC Lead	
Alex	Allwood			Derriford Hospital	England	UKNC Lead	
Vijaya	Hebbar			Diana Princess of Wales Hospital	England	UKNC Lead	
Nigel	Brooke			Doncaster Royal Infirmary	England	UKNC Lead	
Claire	Hollinsworth			Dorset County Hospital	England	UKNC Lead	
Toria	Klutse			East Surrey Hospital	England	UKNC Lead	
Clare	Sturdy			Epsom General Hospital	England	UKNC Lead	
Sathish	Krishnan			Frimley Park Hospital	England	UKNC Lead	
Maria	Hadjicosta			Furness General Hospital	England	UKNC Lead	
Sabyasachi	Chowdhury			George Eliot Hospital	England	UKNC Lead	
Shyam	Bhakthavalsala			Gloucester Royal Hospital	England	UKNC Lead	
Daniel	Dogar			Good Hope Hospital	England	UKNC Lead	
Girish	Gowda			Great Western Hospital	England	UKNC Lead	
Karen	Turnock			Guy's & St Thomas' Hospital	England	UKNC Lead	
Patricia	Gilbertson			Harrogate District Hospital	England	UKNC Lead	
Cath	Seagrave			Hereford County Hospital	England	UKNC Lead	
Tristan	Bate			Hillingdon Hospital	England	UKNC Lead	
Hilary	Dixon			Hinchingbrooke Hospital	England	UKNC Lead	
Narendra	Aladangady			Homerton Hospital	England	UKNC Lead	
Hassan	Gaili			Hull Royal infirmary	England	UKNC Lead	
Matthew	James			Ipswich Hospital	England	UKNC Lead	
M	Lal			James Cook University Hospital	England	UKNC Lead	
Oluseun	Tayo			James Paget Hospital	England	UKNC Lead	
Abraham	Isaac			Kettering General Hospital	England	UKNC Lead	
Carolina	Zorro			Kings College Hospital	England	UKNC Lead	
Dhaval	Dave			King's Mill Hospital	England	UKNC Lead	

Supplemental Online Content: Nonauthor Collaborators
*First name, last name, and suffix (if applicable) are required and will appear in PubMed.

***First Name and Middle Initial(s)**	***Last Name**	***Suffix (eg, Jr, III)**	Academic Degrees	Institution	Location (city, state/province, country)	Role or Contribution, eg, chair, principal investigator	Group (if more than 1 Group listed in the byline) and/or Subgroup (eg, Steering Committee)
Jonathan	Filkin			Kingston Hospital	England	UKNC Lead	
Savi	Sivashankar			Lancashire Women and Newborn Centre	England	UKNC Lead	
Hannah	Shore			Leeds General Infirmary	England	UKNC Lead	
Jo	Behrsin			Leicester General Hospital and Leicester Royal Infirmary	England	UKNC Lead	
Michael	Grosdenier			Leighton Hospital	England	UKNC Lead	
Ruchika	Gupta			Lincoln County Hospital	England	UKNC Lead	
Ather	Ahmed			Lister Hospital	England	UKNC Lead	
Nim	Subhedar			Liverpool Women's Hospital	England	UKNC Lead	
Jennifer	Birch			Luton & Dunstable Hospital	England	UKNC Lead	
Surendran	Chandrasekara n			Macclesfield District General Hospital	England	UKNC Lead	
Ashok	Karupaiah			Manor Hospital (Walsall)	England	UKNC Lead	
Ghada	Ramadan			Medway Maritime Hospital	England	UKNC Lead	
I	Misra			Milton Keynes General Hospital	England	UKNC Lead	
Chris	Knight			Musgrove Park Hospital	England	UKNC Lead	
Richard	Heaver			New Cross Hospital	England	UKNC Lead	
Mohammad	Alam			Newham General Hospital	England	UKNC Lead	
Prakash	Thiagarajan			Nobles Hospital	England	UKNC Lead	
Priya	Muthukumar			Norfolk & Norwich University Hospital	England	UKNC Lead	
Tiziana	Fragapane			North Devon District Hospital	England	UKNC Lead	
Bivan	Saha			North Manchester General Hospital	England	UKNC Lead	
Cheentan	Singh			North Middlesex University Hospital	England	UKNC Lead	
Nick	Barnes			Northampton General Hospital	England	UKNC Lead	

Supplemental Online Content: Nonauthor Collaborators
*First name, last name, and suffix (if applicable) are required and will appear in PubMed.

*First Name and Middle Initial(s)	*Last Name	*Suffix (eg, Jr, III)	Academic Degrees	Institution	Location (city, state/province, country)	Role or Contribution, eg, chair, principal investigator	Group (if more than 1 Group listed in the byline) and/or Subgroup (eg, Steering Committee)
Sangeeta	Tiwary			Northumbria Specialist Emergency Care Hospital	England	UKNC Lead	
Richard	Nicholl			Northwick Park Hospital	England	UKNC Lead	
Dush	Batra			Nottingham City Hospital and Nottingham University Hospital (QMC)	England	UKNC Lead	
Victoria	Nesbitt			Ormskirk District General Hospital	England	UKNC Lead	
Amit	Gupta			Oxford University Hospitals, John Radcliffe Hospital	England	UKNC Lead	
Katharine	McDevitt			Peterborough City Hospital	England	UKNC Lead	
Ruchika	Gupta			Pilgrim Hospital	England	UKNC Lead	
David	Gibson			Pinderfields General Hospital	England	UKNC Lead	
Peter	Mcewan			Poole General Hospital	England	UKNC Lead	
Sanath	Reddy			Princess Alexandra Hospital	England	UKNC Lead	
Mark	Johnson			Princess Anne Hospital	England	UKNC Lead	
Cassie	Lawn			Princess Royal Hospital	England	UKNC Lead	
Patricia	Cowley			Princess Royal Hospital Telford	England	UKNC Lead	
Rashmi	Gandhi			Princess Royal University Hospital	England	UKNC Lead	
Charlotte	Groves			Queen Alexandra Hospital	England	UKNC Lead	
Lidia	Tyszczuzk			Queen Charlotte's Hospital	England	UKNC Lead	
Shilpa	Ramesh			Queen Elizabeth Hospital, Gateshead	England	UKNC Lead	
Salamatu	Jalloh			Queen Elizabeth Hospital, King's Lynn	England	UKNC Lead	
Julia	Croft			Queen Elizabeth Hospital, Woolwich	England	UKNC Lead	
Bushra	Abdul-Malik			Queen Elizabeth the Queen Mother Hospital	England	UKNC Lead	

*First name, last name, and suffix (if applicable) are required and will appear in PubMed.

*First Name and Middle Initial(s)	*Last Name	*Suffix (eg, Jr, III)	Academic Degrees	Institution	Location (city, state/province, country)	Role or Contribution, eg, chair, principal investigator	Group (if more than 1 Group listed in the byline) and/or Subgroup (eg, Steering Committee)
Dominic	Muogbo			Queen's Hospital, Burton on Trent	England	UKNC Lead	
Ambalika	Das			Queen's Hospital, Romford	England	UKNC Lead	
Khalid	Mannan			Queen's Hospital, Romford	England	UKNC Lead	
Rajiv	Chaudhary			Rosie Maternity Hospital, Addenbrookes	England	UKNC Lead	
Soma	Sengupta			Rotherham District General Hospital	England	UKNC Lead	
Christos	Zipitis			Royal Albert Edward Infirmary	England	UKNC Lead	
Kemy	Naidoo			Royal Berkshire Hospital	England	UKNC Lead	
Dinakar	Seshadri			Royal Bolton Hospital	England	UKNC Lead	
Chris	Warren			Royal Cornwall Hospital	England	UKNC Lead	
Nigel	Ruggins			Royal Derby Hospital	England	UKNC Lead	
Chrissie	Oliver			Royal Devon & Exeter Hospital	England	UKNC Lead	
Lucinda	Winckworth			Royal Hampshire County Hospital	England	UKNC Lead	
Joanne	Fedee			Royal Lancaster Infirmary	England	UKNC Lead	
Anitha	Vayalakkad			Royal Oldham Hospital	England	UKNC Lead	
Richa	Gupta			Royal Preston Hospital	England	UKNC Lead	
Julia	Uffindell			Royal Stoke University Hospital	England	UKNC Lead	
Jo	MacLeod			Royal Surrey County Hospital	England	UKNC Lead	
Cassie	Lawn			Royal Sussex County Hospital	England	UKNC Lead	
Rebecca	Winterson			Royal United Hospital	England	UKNC Lead	
Naveen	Athiraman			Royal Victoria Infirmary	England	UKNC Lead	
Muhammad	Khurshid			Russells Hall Hospital	England	UKNC Lead	
Jim	Baird			Salisbury District Hospital	England	UKNC Lead	
Adedayo	Owoeye			Scarborough General Hospital	England	UKNC Lead	
Umapathee	Majuran			Scunthorpe General Hospital	England	UKNC Lead	

Supplemental Online Content: Nonauthor Collaborators
*First name, last name, and suffix (if applicable) are required and will appear in PubMed.

*First Name and Middle Initial(s)	*Last Name	*Suffix (eg, Jr, III)	Academic Degrees	Institution	Location (city, state/province, country)	Role or Contribution, eg, chair, principal investigator	Group (if more than 1 Group listed in the byline) and/or Subgroup (eg, Steering Committee)
Richard	Lindley			Sheffield Children's Hospital	England	UKNC Lead	
Vineet	Vineet Gupta			Southend Hospital	England	UKNC Lead	
Faith	Emery			Southmead Hospital	England	UKNC Lead	
Madhavi	Parvathareddy			Southmead Hospital	England	UKNC Lead	
Donovan	Duffy			St George's Hospital	England	UKNC Lead	
Salim	Yasin			St Helier Hospital	England	UKNC Lead	
Hannah	Shore			St James University Hospital	England	UKNC Lead	
Akinsola	Ogundiya			St Mary's Hospital, IOW	England	UKNC Lead	
Lidia	Lidia			St Mary's Hospital, London	England	UKNC Lead	
Arin	Mukherjee			St Mary's Hospital, Manchester	England	UKNC Lead	
Pamela	Cairns			St Michael's Hospital	England	UKNC Lead	
Vennila	Ponnusamy			St Peter's Hospital	England	UKNC Lead	
Victoria	Sharp			St Richard's Hospital	England	UKNC Lead	
Carrie	Heal			Stepping Hill Hospital	England	UKNC Lead	
Sanjay	Salgia			Stoke Mandeville Hospital	England	UKNC Lead	
Imran	Ahmed			Sunderland Royal Hospital	England	UKNC Lead	
Helen	Purves			Tameside General Hospital	England	UKNC Lead	
Porus	Bastani			The Jessop Wing, Sheffield	England	UKNC Lead	
Eleanor	Bond			The Royal Free Hospital	England	UKNC Lead	
Divyen	Shah			The Royal London Hospital - Constance Green	England	UKNC Lead	
Esther	Morris			Torbay Hospital	England	UKNC Lead	
Mithun	Urs			Tunbridge Wells Hospital	England	UKNC Lead	
Giles	Kendall			University College Hospital	England	UKNC Lead	
Puneet	Nath			University Hospital Coventry	England	UKNC Lead	
Igor	Fierens			University Hospital Lewisham	England	UKNC Lead	
Mehdi	Garbash			University Hospital of North Durham	England	UKNC Lead	

Supplemental Online Content: Nonauthor Collaborators
*First name, last name, and suffix (if applicable) are required and will appear in PubMed.

*First Name and Middle Initial(s)	*Last Name	*Suffix (eg, Jr, III)	Academic Degrees	Institution	Location (city, state/province, country)	Role or Contribution, eg, chair, principal investigator	Group (if more than 1 Group listed in the byline) and/or Subgroup (eg, Steering Committee)
Hari	Kumar			University Hospital of North Tees	England	UKNC Lead	
Peter	Curtis			Victoria Hospital, Blackpool	England	UKNC Lead	
Delyth	Webb			Warrington Hospital	England	UKNC Lead	
Sumedha	Bird			Warwick Hospital	England	UKNC Lead	
Sankara	Narayanan			Watford General Hospital	England	UKNC Lead	
Yee Mon	Aung			West Cumberland Hospital	England	UKNC Lead	
Elizabeth	Eyre			West Middlesex University Hospital	England	UKNC Lead	
Tayyaba	Aamir			West Suffolk Hospital	England	UKNC Lead	
Angela	Yannoulias			Wexham Park Hospital	England	UKNC Lead	
Caroline	Sullivan			Whipps Cross University Hospital	England	UKNC Lead	
Ros	Garr			Whiston Hospital	England	UKNC Lead	
Wynne	Leith			Whittington Hospital	England	UKNC Lead	
Shaveta	Mulla			William Harvey Hospital	England	UKNC Lead	
Anna	Gregory			Worcestershire Royal Hospital	England	UKNC Lead	
Edward	Yates			Worthing Hospital	England	UKNC Lead	
Abijeet	Godhamgaonkar			Wythenshawe Hospital	England	UKNC Lead	
Siba	Paul			Yeovil District Hospital	England	UKNC Lead	
Sundeep	Sandhu			York District Hospital	England	UKNC Lead	
Saulius	Satas			Aberdeen Maternity Hospital,	Scotland	UKNC Lead	
Clare	Irving			Borders General Hospital, Melrose	Scotland	UKNC Lead	
Andrew	Eccleston			Dumfries and Galloway Royal Infirmary	Scotland	UKNC Lead	
Tim	Adams			Cross House Hospital, Kilmarnock	Scotland	UKNC Lead	

Supplemental Online Content: Nonauthor Collaborators
*First name, last name, and suffix (if applicable) are required and will appear in PubMed.

***First Name and Middle Initial(s)**	***Last Name**	***Suffix (eg, Jr, III)**	Academic Degrees	Institution	Location (city, state/province, country)	Role or Contribution, eg, chair, principal investigator	Group (if more than 1 Group listed in the byline) and/or Subgroup (eg, Steering Committee)
Dominic	O'Reilly			Forth Valley Hospital, Larbert	Scotland	UKNC Lead	
Bhushan	Bhushan			Ninewells Hospital, Dundee	Scotland	UKNC Lead	
Carolyn	Abernethy			Princess Royal Maternity Hospital, Glasgow	Scotland	UKNC Lead	
P	Van Der Heide			Raigmore Hospital, Inverness	Scotland	UKNC Lead	
Hilary	Conetta			Royal Alexandra Hospital, Paisley	Scotland	UKNC Lead	
AM	Heuchan			The Queen Elizabeth University Hospital Glasgow	Scotland	UKNC Lead	
Ben	Stenson			Simpsons Centre for Reproductive Health, Royal Infirmary of Edinburgh	Scotland	UKNC Lead	
Helen	Rhodes			St John’s Hospital, Livingston	Scotland	UKNC Lead	
Laura	Stewart			Victoria Hospital, Kirkcaldy	Scotland	UKNC Lead	
Lorraine	McGlory			Wishaw General Hospital	Scotland	UKNC Lead	
Arun	Ramachandran			Singleton Hospital	Wales	UKNC Lead	
Abby	Parish			Princess of Wales Hospital	Wales	UKNC Lead	
Anitha	James			The Grange University Hospital	Wales	UKNC Lead	
Ambrose	Onibere			Glan Clwyd Hospital	Wales	UKNC Lead	
Artur	Abelian			Wrexham Maelor Hospital	Wales	UKNC Lead	
Shakir	Saeed			Ysbyty Gwynedd	Wales	UKNC Lead	
Nitin	Goel			University Hospital of Wales	Wales	UKNC Lead	
David	Deekollu			Prince Charles Hospital	Wales	UKNC Lead	
Prem	Pitchaikani			Glangwili General Hospital	Wales	UKNC Lead	
Stan	Craig			Royal Maternity Hospital	Northern Ireland	UKNC Lead	
Sanjeev	Bali			Antrim Area Hospital	Northern Ireland	UKNC Lead	
Nita	Saxena			Ulster Hospital	Northern Ireland	UKNC Lead	

Supplemental Online Content: Nonauthor Collaborators
*First name, last name, and suffix (if applicable) are required and will appear in PubMed.

*First Name and Middle Initial(s)	*Last Name	*Suffix (eg, Jr, III)	Academic Degrees	Institution	Location (city, state/province, country)	Role or Contribution, eg, chair, principal investigator	Group (if more than 1 Group listed in the byline) and/or Subgroup (eg, Steering Committee)
Alison	Verner			Craigavon Area Hospital, Daisy Hill Hospital	Northern Ireland	UKNC Lead	
Damien	Armstrong			Altnagelvin Area Hospital , South West Acute Hospital (SWAH)	Northern Ireland	UKNC Lead	
