# Supplementary material for: Neonatal Morbidities and Hospitalization in the First 2 Years of Life Among Infants Born Very Preterm
Source: JAMA Netw Open. 2025 Sep 3;8(9):e2530123. doi: 10.1001/jamanetworkopen.2025.30123 (PMC12409584; doi:10.1001/jamanetworkopen.2025.30123)
Supplement: Supplement 3. — Data Sharing Statement [file jamanetwopen-e2530123-s003.pdf]

Data Sharing Statement

van Hasselt. Neonatal Morbidities and Hospitalization in the First 2 Years of Life Among Infants Born Very Preterm. *JAMA Netw Open*. Published September 03, 2025.
doi:10.1001/jamanetworkopen.2025.30123

Data
Data available: No

Additional Information
Explanation for why data not available: Data Sharing Statement The National Neonatal Research Database (NNRD) has been created through the collaborative efforts of neonatal services across the country to be a national resource. The NNRD was developed and is maintained and managed at the Neonatal Data Analysis Unit (NDAU) at the Chelsea and Westminster NHS Foundation Trust campus of Imperial College London, led by Professor Neena Modi. Researchers, clinicians, managers, commissioners, and others are welcome to utilise the NNRD and NNRD-AI. The NDAU receives no core funding to support the NNRD or NNRD-AI. For this reason a charge is necessary to cover the costs of maintaining and developing the NNRD/NNRD-AI, data transfer, extraction, cleaning, and storage, and any analyses requested. A Steering Board provides oversight of the NNRD/NNRD-AI. The Steering Board does not provide peer review but ensures the request is legitimate, feasible and in the interests of patients or the public. If the request is approved, the applicant is notified and asked to obtain necessary regulatory approvals. Applicants are asked to confirm that research findings will be published. On confirmation of regulatory approval, all eligible neonatal units are sent a copy of the research protocol, inviting them to participate. In the case of nationally commissioned service evaluations, all neonatal units are notified of the request by the lead organisation. These processes ensure that neonatal units are able to decline participation in research if they wish and are fully informed about national initiatives. If you are thinking of using the NNRD or NNRD-AI for research or health services audit or evaluations, please first read the information available from https://www.imperial.ac.uk/neonatal-data-analysis-unit/neonatal-data-analysis-unit/utilising-the-nnrd carefully, follow the process shown and complete the Health Data Research UK data access request (www.healthdatagateway.org/dataset/67020745-9def-4c6e-b5ac-bb273bd0a20e">https://web.www.healthdatagateway.org/dataset/67020745-9def-4c6e-b5ac-bb273bd0a20e). Admitted Patient Care data are available from NHS England and NHS Wales Informatics Service.
